# Complementary Feeding Caregivers’ Practices and Growth, Risk of Overweight/Obesity, and Other Non-Communicable Diseases: A Systematic Review and Meta-Analysis

**DOI:** 10.3390/nu14132646

**Published:** 2022-06-26

**Authors:** Marcello Bergamini, Giovanni Simeone, Maria Carmen Verga, Mattia Doria, Barbara Cuomo, Giuseppe D’Antonio, Iride Dello Iacono, Giuseppe Di Mauro, Lucia Leonardi, Vito Leonardo Miniello, Filomena Palma, Immacolata Scotese, Giovanna Tezza, Margherita Caroli, Andrea Vania

**Affiliations:** 1 AUSL Ferrara, 44121 Ferrara, Italy; 2 ASL Brindisi, 72023 Mesagne, Brindisi, Italy; giovanni.simeone@gmail.com; 3 ASL Salerno, 84019 Vietri Sul Mare, Salerno, Italy; vergasa@virgilio.it; 4 AULSS 3 Serenissima, 30015 Chioggia, Venice, Italy; mattiadoria@fimp.pro; 5 Department of Pediatrics, Belcolle Hospital, 01010 Viterbo, Italy; cuomoba@gmail.com; 6 Independent Researcher, 84100 Salerno, Italy; gdantonio32@gmail.com; 7 Independent Researcher, 82100 Benevento, Italy; iridedello@gmail.com; 8 ASL Caserta, 81031 Aversa, Caserta, Italy; presidenza@sipps.it; 9 Maternal Infantile and Urological Sciences Department, Sapienza University, 00161 Rome, Italy; lucialeonardi@yahoo.it; 10 Nutrition Unit, Department of Pediatrics, “Giovanni XXIII” Children Hospital, “Aldo Moro” University of Bari, 70126 Bari, Italy; vito.miniello@libero.it; 11 ASL Salerno, 84091 Battipaglia, Salerno, Italy; menapalma3@gmail.com; 12 ASL Salerno, 84022 Campagna, Salerno, Italy; scotese.ped@libero.it; 13 San Bortolo Hospital, 36100 Vicenza, Italy; giovanna.tezza@gmail.com; 14 Independent Researcher, 72021 Francavilla Fontana, Brindisi, Italy; margheritacaroli53@gmail.com; 15 Independent Researcher, 00162 Rome, Italy; andrea.vania57@gmail.com

**Keywords:** complementary feeding, weaning, responsive feeding, non-responsive feeding, baby led weaning, BLISS, growth, overweight, obesity, choking

## Abstract

Several institutions propose responsive feeding (RF) as the caregivers’ relational standard when nurturing a child, from breast/formula feeding onwards. Previous systematic reviews (SRs) on caregivers’ feeding practices (CFPs) have included studies on populations from countries with different cultures, rates of malnutrition, and incomes, whereas this SR compares different CFPs only in healthy children (4–24 months) from industrialized countries. Clinical questions were about the influence of different CFPs on several important outcomes, namely growth, overweight/obesity, risk of choking, dental caries, type 2 diabetes (DM2), and hypertension. The literature review does not support any Baby Led Weaning’s or Baby-Led Introduction to SolidS’ (BLISS) positive influence on children’s weight–length gain, nor their preventive effect on future overweight/obesity. RF-CFPs can result in adequate weight gain and a lower incidence of overweight/obesity during the first two years of life, whereas restrictive styles and coercive styles, two kinds of non-RF in CF, can have a negative effect, favoring excess weight and lower weight, respectively. Choking risk: failure to supervise a child’s meals by an adult represents the most important risk factor; no cause–effect relation between BLW/BLISS/RF/NRCF and choking could be found. Risks of DM2, hypertension, and caries: different CFPs cannot be considered as a risky or preventive factor for developing these conditions later in life.

## 1. Introduction

Until the 1980s, the main focus of complementary feeding (CF) research was on infant nutrition. Later on, in the 1990s, new models were proposed that were mainly based on the strategies and behaviors to be adopted by parents at mealtimes to encourage semi-solid food intake by their child [1,2,3,4].

Subsequently, research efforts started to focus on the hypothesis that the different infant and young child feeding styles might positively or negatively influence the metabolic processes underlying both the physical and neuropsychological development of children [5,6].

Since the early 2000s, a CF practice referred to as baby led weaning (BLW) has been established, especially in some Anglo-Saxon countries (Great Britain and New Zealand) [1,7]. BLW was developed from the simple observation by the British midwife Gill Rapley of the behavioral responses of five infants when offered pieces of food easy for the babies to pick up at family mealtimes. No nutritional analysis was included in these studies.

As a general rule, with BLW, infants are offered the same food the rest of the family is eating in pieces in a size and shape that they can pick up and feed themselves, independently. The parent decides what to offer, but it is the baby who decides what he/she will eat (of the food offered), how much, and how quickly.

BLW also differs from conventional CF methods in that it does not include baby food, whether commercial or home-prepared. One of the objectives of BLW is to allow children to continue their experience of breastfeeding, whereby they have already been exposed to a variety of tastes and flavors that will help them to more readily accept complementary foods. What is more, BLW, as is currently recommended in other CF models, includes a wide variety of foods (fruit, vegetables, meat, cheese, hard-boiled eggs, bread or bread toast, pasta, fish) [7,8,9,10] from the start, but these foods are cut and prepared in such a way that children can manipulate and eat them on their own, without the help of a caregiver or cutlery.

Therefore, the core principle of BLW is the child’s manipulation of the food offered to him or her because, according to the inventor of BLW, this could lead to benefits in terms of the child getting familiar with and starting to like different kinds of food.

This implies the following:

1. Parent modelling role is more limited: caregivers can continue to eat separately and in different ways;
2. The child is also allowed to eat outside family mealtimes, as the request/response element is missing;
3. The assumption is that the child is “offered” a food that he or she can handle and does not “request” it as a result of imitating other family members at mealtimes;
4. The risk does exist to allow for feeding the child with a poor/unbalanced diet characterized by a lack of essential nutrients, because in the early stages, not all foods rich in these nutrients (e.g., meat and fish) can be easily manipulated or eaten by the child.

Finally, with BLW, mothers are less likely to put pressure on their babies by forcing them to eat or restricting their food intake, i.e., two non-responsive feeding (NRF) practices.

Some studies have correlated BLW with a risk of macronutrient deficiencies and particularly of key micronutrients (e.g., iron, zinc, and fat-soluble vitamins) in the second half of a child’s first year of life, as well as with an increased risk of choking [11,12,13,14,15].

On the basis of these studies, a group of New Zealand investigators developed and proposed a controlled model of BLW, referred to as BLISS (Baby-Led Introduction to SolidS), where the basic principles of BLW are kept, but they also encourage parents to offer three different types of food at each meal: an iron-rich food (red meat or iron-fortified infant cereals), an energy-rich food, and a fiber-rich food such as a fruit or vegetable [16]. In addition, BLISS also provides information on how food should be prepared in order to reduce the risk of choking.

More recently, both the World Health Organization (WHO) [17] and the American Academy of Pediatrics (AAP) [18] have proposed responsive feeding (RF) as the caregiver–child relational standard to be adopted when feeding a child, from the early stages of breastfeeding or formula feeding, through to the CF period and beyond. RF has been indicated as a possible driver in the implementation of health promotion programs in industrialized countries, where the almost unlimited availability of food can expose even children to the risk of developing non-communicable diseases (NCDs) [19,20].

RF enhances both active behavior by the child and an appropriate response by the caregiver. In this SR, RF-based CF is referred to as responsive complementary feeding (RCF).

The offer of food occurs only as a response to hunger cues from the infant, who begins to show interest in solid foods, and stops when the infant stops demanding food. Food is offered at the times and in the ways, textures, and quantities that are most appropriate to the infant’s stage of psycho-neuro-motor and physical development.

Thus, the key element is once again the request from the child, as opposed to the BLW, where priority is given to the absolute self-dependence of the child with little attention to relational aspects. 

The second key aspect is that the family must necessarily adopt a diet that is nutritionally appropriate, both for the adults and children.

A key issue of RCF is that there is no formal definition of the percentages of the various macronutrients, but families are given freedom of choice within eating styles deemed to be healthy [21].

Non-responsive feeding (NRF), which in this SR, if adopted in the CF period, is referred to as non-responsive complementary feeding (NRCF), is based on a non-synchronous caregiver–child relationship. The hunger or satiety cues from the child are not recognized/taken into account by the caregiver, who rather controls the feeding process according to his/her feelings or convictions, forcing the infant to eat or restricting his/her food intake or using food for other purposes or even being completely uninvolved.

Finally, the CF that is traditionally used in many countries, the traditional complementary feeding (TCF), relies on the use of mainly commercial infant food products that are progressively introduced according to standardized schedules. Indicative portions of these foods are recommended by pediatricians or other health professionals.

Table 1 summarizes the main characteristics of the different CF methods.

### 1.1. Why This Systematic Review Is Important

The systematic reviews (SRs) published to date in this field of research have included studies conducted on populations from countries in different geographic regions, with very different cultures and different rates of early-life malnutrition and income levels.

In contrast, this SR is specifically focused on comparative assessments of the different styles of CF adopted in a population of healthy children aged 4–24 months in industrialized countries.

### 1.2. Objectives

The aim of this SR is to give an answer to some clinical questions concerning the influence of different caregivers’ feeding practices (CFPs), i.e., the strategies or behaviors parents use to direct child eating [6] on the risk of choking, as well as on the development of the following outcomes later in life: growth, risk of overweight, and/or obesity.

Additional outcome(s): risk of developing dental caries, DM2, and hypertension later in life.

### 1.3. Key Questions

- Can the BLW/BLISS method during CF influence, either positively or negatively, infant weight–length gain?
- Can the BLW/BLISS method during CF influence, either positively or negatively, the development of overweight/obesity?
- Can RF during the CF period (responsive complementary feeding—RCF) influence, either positively or negatively, physical growth?
- Can non-responsive feeding during the CF period (non-responsive complementary feeding—NRCF) influence, either positively or negatively, physical growth?
- Can RCF influence the development of overweight and obesity?
- Can NRCF influence the development of overweight and obesity?
- Do the different caregivers’ feeding practices (CFPs) during the CF period result in different risks of choking?
- Can RCF influence the development of type 2 diabetes mellitus (DM2)?
- Can TCF influence the development of DM2?
- Can RCF influence the development of hypertension?
- Can TCF influence the development of hypertension?
- Can RCF influence the development of dental caries?
- Can TCF influence the development of dental caries?

The questions, structured according to PICO, are reported in Supplementary File S1.

## 2. Materials and Methods

The protocol of this SR was registered on PROSPERO, International Prospective Register of Systematic Reviews CRD42021259486 (Available online: https://www.crd.york.ac.uk/prospero/n°CRD42021259486, accessed on 15 March 2022) [22].

The formulation of clinical questions, the search, the analysis of the scientific evidence with specific tools [23,24,25,26], and the GRADE method [27,28,29] have been previously described [30].

### 2.1. Design of the Studies Included

The studies included were as follows:

- Randomized controlled trials (RCTs) and controlled trials (CTs) in which the effect of the caregivers’ feeding styles could be accurately assessed as an experimental intervention;
- Observational studies (cohort studies, longitudinal studies, case-control studies, and cross-sectional studies) in which this effect could be evaluated as an exposure factor while taking into account possible confounding factors.

To improve bibliographic search resources and to obtain further elements for the evaluation of the studies included in this SR, evidence-based guidelines, government publications, and already published SRs considered to be of good quality were also selected and evaluated.

### 2.2. Population

Healthy, term-born, normal birth weight, and breastfed and/or formula-fed children, aged 4-24 months or older for short- and long-term outcomes, residing in Western industrialized countries.

### 2.3. Intervention(s), Exposure(s)

Caregivers’ feeding practices (CFPs): BLW, BLISS, and RCF.

### 2.4. Comparator(s)/Control

Different caregivers’ feeding practices: TCF and NRCF.

### 2.5. Inclusion Criteria

Studies focusing on the influence of different CFPs on the outcomes under consideration were deemed to meet the eligibility criteria to be included in this SR: retrospective observational studies where other different CFPs had been observed over time (exposure).

Prospective controlled studies conducted in Western industrialized countries where the intervention consisted of educational sessions addressed to mothers or caregivers. Comparative studies with traditional CF and with specific non-responsive CF methods, when specifically defined, were considered to meet eligibility criteria.

The educational interventions for caregivers reported in the controlled studies could

- Be addressed to mothers or to the whole family;
- Start before the birth of the child and then continue in the first months or years of life of the child, or start after the birth and continue in the first months or years of life of the child;
- Be associated or not associated with any recommendations on the intake of specific foods rich in macro- and micro-nutrients.

Other inclusion criteria were

- The presence of objective outcome indicators (measurement of weight (W), length/height (L/H), head circumference (HC), etc.), with the exclusion of studies in which the outcome was made up only of behavioral or psychological indices of the individual elements of the mother/child dyad, or of the dyad itself;
- a ≥ 6 month follow-up at least introducing CF.

### 2.6. Exclusion Criteria

Studies conducted on populations with characteristics different from those established in the PICOs (e.g., children living in developing countries, preterm infants, low birth weight infants, children who developed peri-neonatal diseases, and children with chronic diseases) were excluded. In addition, studies and SRs published in recent years dealing with interventions and educational programs carried out in developing countries with the aim of reducing mortality and improving feeding techniques, thereby enhancing nutritional parameters and growth, were also excluded.

Studies with methodological biases likely to affect the confidence in the results obtained were also excluded [31,32].

### 2.7. Main Outcomes

- General growth parameters assessed in prospective differential terms (different increase in W or L over time) or assessed at a specific time point (with differing frequencies of weights and lengths in the populations being compared: W, L, W/L z-score ratio, BMI, BMI z-score (BMIz);
- Risk of NCDs (overweight/obesity, DM2, and hypertension);
- Risk of choking;
- Risk of dental caries.

### 2.8. Keywords and Search Strategy

See Supplementary File S1.

### 2.9. Measures of Effect

The standard methods of the Cochrane Review Group were used to synthesize the data; the effects have been reported as risk ratio (RR), risk difference (RD) with 95% confidence intervals (CIs) for categorical data, and as mean difference (MD) or standardize main difference (SDM) (95% CIs) for continuous data [30].

### 2.10. Studies Selection

Studies Selection. Risk of Bias (Quality) Assessment. Missing Data. List of the studies excluded with relevant reasons. Data Extraction (Selection and Coding) See Supplementary Files S2 and S3.

The selection process and the assessment of the overall methodological quality and of the quality of the individual studies, including missing data handling and data extraction, were conducted by at least two mutually blind authors, as already reported [30].

The SRs and the studies were evaluated using specific tools [24,25,26], as previously described [30].

The following data were extracted from the studies: author, year of publication, study design, objective of the study, country, sample, healthy or pathological condition, age, type of intervention, period of follow-up, results, main conclusions of the study, and financing.

### 2.11. Assessment of Heterogeneity

The chi-square test was used to test heterogeneity; to quantify the percentage of total variation across studies due to heterogeneity, the I2 index was calculated. The fixed-effect model was used for meta-analysis when enrolled infants and interventions were similar and no significant heterogeneity was found. The sources of heterogeneity were explored by performing a subgroup analysis.

### 2.12. Strategy for Data Synthesis

A meta-analysis was conducted when data merging was possible. In the presence of significant statistical heterogeneity, data were combined using the random effect model. The Mantel–Haenszel analysis was used for the dichotomous scores and inverse variance for the continuous scores.

### 2.13. Publication Bias Assessment

When possible (number of studies included in the meta-analysis ≥10), the publication bias was assessed by examining the degree of asymmetry of a funnel plot.

### 2.14. Software

The software RevMan 5.4.1 was used to report the quality of RCTs, perform the meta-analysis of the data entered, and present the results graphically [33].

The GRADEpro GDT software, developed by the GRADE Working Group, was used to grade the overall quality of evidence and the related tables [34].

## 3. Results

### 3.1. Can the BLW/BLISS Method during CF Influence, Either Positively or Negatively, Infant Weight–Length Gain?

No evidence-based GLs with a specific focus on the relationship between BLW during the CF period and child growth were found.

Two relevant SRs were included, one of moderate quality [10], another of low quality [35].

The two cross-sectional observational studies relevant to the clinical question included in the RS by D’Auria et al. are of low [12] and moderate [21] methodological quality, respectively, for the following:

- Recruitment of the mothers who intended to adopt BLW on a voluntary basis;
- Uncertainty of weight measurement by parents, with unspecified frequency;
- Significant drop-out rate during the period of observation;
- Non-homogeneous intervention and control groups in terms of subject age.

Both studies showed, although at different ages (20–78 months for the first and 18–24 months for the second), a higher frequency of underweight children in the BLW group and a higher frequency of overweight/obese children in the group fed with traditional British food.

Only one observational study was included in the SR by Martinón-Torres et al. [36]. In this study, of moderate methodological quality, no significant differences in L were observed between children who received BLW feeding and children who received traditional or mixed feeding practices (*p* = 0.437), while regarding the achievement of adequate weight, the children with BLW were disadvantaged (OR = 0.21, 95% C.I. 0.06–0.77). These data, however, were not confirmed by our meta-analysis on the results of the three studies (OR = 1.36, 95% C.I. 0.18–10.51) (Supplementary File S4, Analysis 1.1). A major bias in this work was that the exact age at which the growth parameters were measured was not defined.

In a randomized trial of low methodological quality, included in the SR by D’Auria et al. [37], the intervention consisted of the BLISS method. No significant differences in BMI and BMIz were observed in the trial in question between children fed with the BLISS approach and those fed traditionally at both 12 and 24 months of age.

An RCT of low methodological quality, published at a later date and included in the RS by Martinòn-Torres et al., was also included [38]. In this trial, conducted in Turkey on 302 children, the children in the intervention group were also fed with the BLISS approach. The authors of this trial demonstrated a more rapid weight gain from 6 to 12 months (*p* = 0.001) in the children fed traditionally compared with those fed with the BLISS approach, while no significant differences were observed between the two groups in the same time interval in terms of an increase in length and head circumference, as well as in the absolute values of these two parameters.

The results of the RCTs on the BLISS approach, partially conflicting with each other, are not comparable with those of the observational studies on BLW, because of the difference in the study design and also because the BLISS approach aims to provide parents with nutritional training/guidance regarding how to prepare the individual meals of their children.

### 3.2. Can the BLW/BLISS Method during CF Influence, Either Positively or Negatively, the Development of Overweight/Obesity?

**Figure 1:**
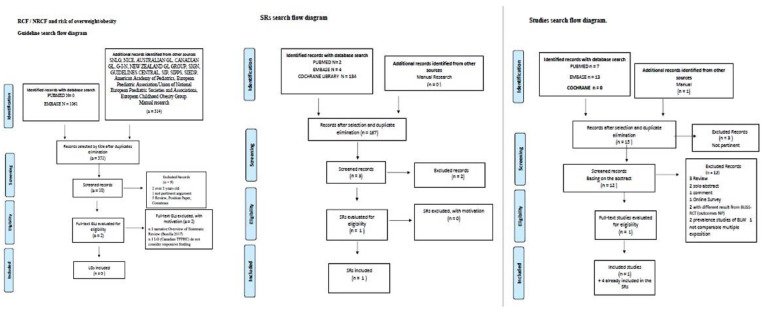
BLW/BLISS and risk of overweight/obesity. Flow diagram of the guidelines, SRs, and studies search.

The ESPGHAN Position Paper on CF [8] points out that BLW, because the clinical studies published to date were observational, is biased by a marked paucity of evidence on obesity prevention and on an improvement of parental responsiveness at mealtimes (Figure 1).

Two SRs have been included [10,35], which include both observational studies on BLW and randomized trials based on the BLISS model.

Three observational studies on BLW were included that reported obesity outcome data, but with a low methodological quality [12,36] and moderate methodological quality [21].

The results on the correlation between BLW and weight outcomes are conflicting. The meta-analysis of the results shows a lower risk of obesity/overweight in the BLW group (OR = 0.37, 95% C.I. = 0.25–0.55, *p* = 0.0001) (Supplementary File S4, Analysis 1.2), but with an overall very low quality of evidence due to voluntary recruitment of mothers intending to use the BLW, uncertainty in weight measurement that was entrusted to parents with unspecified frequency, and significant loss of data during the observation period.

Two low-quality RCTs with overweight as one of the outcome indicators were included in this SR [37,38]. In the first study, no significant correlation was found between BLISS feeding and BMI levels at 12 and 24 months of age.

In the RCT by Dogan et al., a higher weight at 12 months (*p* < 0.001) was observed together with a faster weight gain from 6 to 12 months (*p* = 0.001) and an excessive weight to length ratio (24 vs. 0 children with BMIz greater than 1.5) in children receiving TCF compared with those receiving BLISS, but the results of the meta-analysis on the aggregated data from the two RCTs do not show statistically significant differences (BLISS group: RR = 0.15, 95% C.I. = 0.00–17.79, *p* = 0.44).

The summary of findings for the main comparisons is shown in Table 2.

### 3.3. Can RF during the CF Period (Responsive Complementary Feeding—RCF) Influence, Either Positively or Negatively, Physical Growth?

- Can non-responsive feeding during the CF period (non-responsive complementary feeding—NRCF) influence, either positively or negatively, physical growth?

A secondary literature search allowed for identifying an overview of SRs [39] of good quality, where recommendations of varying strength grades are included. Only two of these recommendations specifically address the feeding styles adopted by caregivers during the early years of life.

In the first recommendation, RF is mentioned in a generic way and not in direct relation to the CF period, as a possible preventive intervention against excessive weight gain thanks to the “*awareness and recognition of the child’s hunger and satiety cues by parents*”.

In the second one, some coercive NRF practices, including pressure to eat and restriction of food intake, are mentioned as counterproductive practices because they would promote wrong eating behaviors and lead to an increase in body weight.

The most recent SR on feeding in the first years of life that is quite comprehensive and of moderate methodological quality is that by Spill et al. [6]. This SR is focused specifically on the influence of CFPs on body weight and size indicators, and also includes observational studies (all but one of which are prospective cohort studies), which are important types of studies for the analysis of feeding-related variables over time.

Of the studies reported in the SR that are also relevant to this clinical question, we included two RCTs and five observational studies.

The first RCT included five papers with a follow-up at 14-60 months, but only the first paper was included in this SR [13], because the results at later ages were biased by a dropout rate of more than 20%, which is not considered acceptable under the methodological evaluation criteria of this SR.

In this paper [13], of good quality, the intervention consisted of six group training sessions, starting at 4 months of age, on various early childhood feeding topics, including CRF; however, the control group had free access to the usual pediatric counseling services.

At 13 months of age, the children in the intervention group were observed to have a lower BMIz score compared with the children in the control group (mean ± DS = 0.23 ± 0.93 and 0.42 ± 0.85, respectively; *p* = 0.01).

In the most recent RCT, INSIGHT [40], of low methodological quality, the intervention was conducted on a group of 279 “primiparous mother/infant” dyads who received repeated home visits targeted to specific RF briefings conducted by caregivers, from 1 to 10 months of age of the children in question. On the other hand, the control group received generic instructions on the quality, quantity, and timing of the meals, as well as training on recognizing children’s nutritional needs. At 1 year of age, the children in the intervention group had a lower WLZ score (*p* = 0.04).

Four observational studies were of moderate quality [41,42,43,44] and one of low quality [45].

These prospective cohort studies that rely on similar, but not overlapping methodology, have evaluated, at different age intervals, including the CF period, the effects on the growth of different CFPs, both RCF and NRCF (pressure to eat, maternal response to food refusal, indulgent, authoritative, restriction, concern about under/overweight, and food to soothe).

The results, which are reported in detail in Supplementary File S3, are thus difficult to compare, but all confirm that each CFP has significant effects on growth.

The literature search for any studies published after the end of the SR literature search by Spill et al. (1 January 2017) identified one relevant RCT of low quality, in which the results of the INSIGHT study were published at three years of age. At this age, only the difference in terms of BMIz persisted between the two groups. This difference had a very low statistical significance but was not clinically relevant (absolute difference: −0.28 in the active group; 95% C.I. −0.53–−0.01; *p* = 0.04) [46].

### 3.4. Can RCF Influence the Development of Overweight and Obesity?

Can non-responsive complementary feeding (NRCF) influence the development of overweight and obesity?

**Figure 2:**
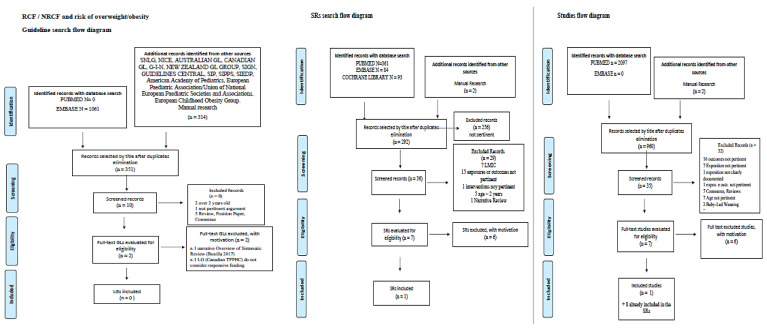
RCF/NRCF and risk of overweight/obesity. Flow diagram of the guidelines, SRs, and studies search.

No evidence-based GLs specifically focused on RCF or NRCF have been found in the last 5 years (Figure 2).

Most of the GLs potentially relevant to the clinical questions on the effects of the diverse CFPs are focused on obesity management in general and obesity prevention.

In the latest ESPGHAN position paper on CF, RCF in the first two years of life is indicated as a possible tool to prevent obesity later in life [8], based on a 2016 SR [47] aimed at identifying the most significant risk factors for obesity. This SR, of a low methodological quality, had overweight and obesity as outcome indicators at the age of 7 years as inclusion criteria and included 27 RCTs. Of these, only three consisted of purely nutritional interventions [14,15,48], while the other 24 included educational/behavioral multiple component interventions (nutrition education and, in general, promotion of appropriate parental feeding styles at mealtimes, promotion of maternal “responsiveness” to the child’s hunger and satiety cues, and parental education on common food neophobia in children). The intervention programs with the best results for overweight, at least in the short term, were those that included motivational approaches or those that focused on the behavior of the child and of the parent, in particular on boosting maternal responsiveness.

In the last 5 years, five relevant SRs have been published, one of which has been included in this SR [6], and from which six studies have been selected: two RCTs [13,40], one CT [49], and three cohort studies [50,51,52].

In the NOURISH study [13] at 13 months of life, children in the intervention group had lower BMIz than children in the control group (mean ± SD = 0.23 ± 0.93 and 0.42 ± 0.85, respectively; *p* = 0.01).

In RCT INSIGHT [40], the number of overweight children in the intervention group, at 1 year of age, was found to be significantly lower than that of children in the control group (*p* = 0.05), with borderline significance.

In the non-randomized controlled trial by Machuca et al. [49], of good quality, the mothers who refused to join the group of active intervention consisting of prolonged group meetings starting at 1 month of age made up the control group. In this study, children in the intervention group were less likely to be overweight at 2 years of age, according to the CDC BMI criteria and percentiles [53].

Among the observational studies, only one cohort study, of low quality, focused on RF and showed non-significant differences in weight outcomes at 18 months [50].

In contrast, the NRF results appeared to be inconsistent or non-significant when adjusted for the various confounding factors in the study of Thompson et al. [50], as well as in the good quality studies of Rifas-Shiman et al. [51] and Lumeng et al. [52].

As far as restrictive style is concerned, the SR by Spill et al. included two studies [44,54] excluded from this SR because they did not include overweight and obesity as outcomes.

The search for studies published after the ending date of the bibliography in the latest SR included [6] led to the inclusion of only two relevant papers, one of which has been included [46], which reports data from the follow-up at age three years of the INSIGHT study. At this age, the percentage of overweight/obesity was significantly lower in the intervention group compared with the control group (for overweight 11.4% vs. 20.8% and obesity 0.8% vs. 8.3%, respectively).

The summary of findings for the main comparisons is shown in Table 3 and Table 4.

### 3.5. Do the Different Caregivers’ Feeding Practices (CFPs), BLW, BLISS, RCF, and NRCF, during the CF Period Result in Different Risks of Choking?

The WHO report on CF [17] and the ESPGHAN position paper [8] did not explore the risk of choking during CF or any cause−effect relationship between choking and the different CFPs.

The Italian Ministry of Health, the Canadian Pediatric Society, and the American Academy of Pediatrics [55,56,57] have published documents providing guidance on how to adequately prepare food and on what behaviors caregivers should adopt during children’s meals, regardless of the age, stage of psychomotor development of the children in question, and of the CFP adopted.

An SR was found [10], with moderate methodological quality, from which two observational studies were selected as relevant to this question, and where exposure was accounted for by BLW [58,59] and one RCT in which the intervention was the BLISS approach [60], and all studies that reported results related to the outcome “choking”.

No statistically significant differences in the rate of choking events were observed between TCF-fed infants and BLW/BLISS-fed infants.

The search for studies published after the SR by D’Auria et al. [10] led to the identification of three relevant studies.

In the first of the cross-sectional studies, conducted in New Zealand [61], of low quality, with surveys based on a simple online questionnaire administered to parents with children 3 years of age, severe choking events were found to be approximately twice as frequent in TCF-fed infants compared with BLW-fed infants (one choking event: 12/876 TCF group vs. 1/155 BLW; non-significant difference (*p* = 0.46).

In the second, conducted in Turkey [36], no significant difference in the risk of choking between the BLW and TCF groups was found from the findings of a questionnaire administered to children of an unspecified age (*p* = 0.855).

In the other study, an RCT of good methodological quality [38], the intervention adopted was BLISS. However, choking and gagging, which were considered as secondary outcomes of the study, had a major detection bias as they were self-reported through a weekly telephone interview. The rate of choking and gagging events was not found to be significantly different between the two groups.

Finally, no SRs or studies relevant to the risk of choking were found that included NRCF or RCF approaches other than those specifically defined as BLW or BLISS.

### 3.6. Can RCF Influence the Development of DM2?

- Can TCF influence the development of DM2?

The systematic search for any GLs that could be relevant to the clinical question did not lead to any results from the past five years. The most important documents either did not deal with prevention specifically or dealt with prevention exclusively from a nutritional perspective.

The search for secondary evidence in the last 10 years did not yield any relevant results.

The search for original studies, which was carried out without time limits, did not identify any studies on the influence of CFPs during CF focused on the risk of developing DM2 later in life.

### 3.7. Can RCF Influence the Development of Hypertension?

No GLs relevant to the clinical question were found in the last 5 years. The literature studies deal with cardiovascular disease prevention exclusively through diet and physical exercise [62].

Educational and intervention programs for the prevention of hypertension, mainly targeted at children and adolescents with risk factors, including DASH [63] and CHILD 1 [64], are exclusively focused on diet nutritional aspects and not on CFPs.

The search for secondary evidence over the last 10 years, as well as the search for primary studies without time limits, did not find any studies on the influence of CFPs during CF and on the risk of developing hypertension later in life.

### 3.8. Can RCF Influence the Development of Dental Caries?

- Can TCF influence the development of dental caries?

None of the four relevant national and international documents [65,66,67,68] found by searching scientific societies’ websites and LG databases focused on the relationship between the development of dental caries and CFPs during the CF period.

The search for SRs identified only one SR that included four observational studies on infant feeding practices, but it was then excluded because the elements considered were not of a behavioral/relational nature and also because the references of the SR in question were from before the time limit adopted in this SR, i.e., 10 years, [69].

The database search for primary clinical trials conducted on populations of pediatric age without time limits found 708 trials, 66 of which included data observation or preventive intervention during CF and had the development of dental caries during childhood as an outcome. None of these trials explored the possible influence of the different CFPs, but only the influence of a number of factors related to feeding behaviors, including breastfeeding, breastfeeding beyond one year of age, breastfeeding at night, formula bottle-feeding, consumption of sweetened beverages, snacks, and CF introduction.

## 4. Discussion

This SR was conducted with the aim of supporting the recommendations of the section on CFPs of the Italian study on CF as a tool for the prevention of NCDs [30].

The latest WHO [17] and ESPGHAN [8] positions recommend RF and RCF as the best relational styles for infant feeding. However, these publications are not updated. In this SR, the evidence-based literature search has been updated to September 2021.

By making decisions on mealtimes, on the quality and quantity of food to be offered, the relational style of feeding, and the rules associated with mealtime sharing, parents play a key role in the CF period, and, consequently, on the growth, the development of the food preferences, and the regulation of appetite of their children [18,70].

### 4.1. Weight–Length Gain and Development of Overweight and Obesity

#### 4.1.1. BLW/BLISS

There is a considerable number of important biases that affect all the studies that have used BLW as a CFP to improve weight and height gain. In particular, the measurement of anthropometric parameters by parents in the BLW group and by specialist health professionals in the control group in the Townsend study [12] has made it very difficult to accept the results obtained as being reliable. On the other hand, the better height gain in the BLW group has also not been confirmed in Kahraman’s study [36], as this study is also biased by the fact that the age at which the measurements were taken is not specified.

As far as the effects of BLISS on growth are concerned, the data derived from the currently available studies, which are in any case of a low methodological quality, are also inconsistent and do not confirm any better effects of BLISS on growth compared with TCF.

The risk of nutritional deficits must also be considered when assessing growth. In BLW, this risk has been poorly assessed through simple questionnaires on micro- and macro-nutrient intake, food preferences, and food variety during the first 2 years of life. Therefore, there is no conclusive evidence that BLW does not cause nutritional deficiencies.

Finally, it should be noted that the studies focused on BLW and BLISS were conducted in Anglo-Saxon countries with their typical “Western” diets, and thus the results of these studies are unlikely to be transferable to populations with different dietary habits. In other words, a specific dietary pattern can turn out to be an advantage in a country with a traditionally unbalanced diet, while it may be a drawback in populations with a healthy diet and healthy lifestyles.

In addition, the currently available evidence on the risk of overweight/obesity does not support a preventive effect for BLW/BLISS: the results of the studies, all of low quality, are conflicting. The preventive efficacy reported by Dogan et al. [38] is indeed not confirmed by the other studies, nor by the meta-analysis of the aggregated data carried out in this SR.

Thus, from the literature review, it cannot be inferred that BLW or BLISS can positively influence children’s weight–length gain, nor that these two approaches have a preventive effect on the development of overweight and obesity. Finally, the possibility cannot be ruled out that BLW might promote macro- and micro-nutrient deficits.

#### 4.1.2. RCF/NRCF

Currently, RF is reported to be the standard relational style to feed children and to guide their transition to correct eating habits from the early stages of breastfeeding or formula feeding, through the CF period and beyond.

On the other hand, NRF styles, which are characterized by a lack of reciprocity between the caregiver and the child at mealtimes and by various forms of adult control, can cause negative effects on the appropriate development and regulation of appetite and satiety, with the risk of altering weight gain.

However, the scientific literature studies on the possible influence of RCFs and NRCFs on growth are heavily biased and the overall evidence quality is thus low. Furthermore, RCTs investigating the different CFPs rely on a similar, but not an identical methodology and, consequently, it is difficult to compare their results.

In general terms, the methodological problems encountered were the following:

- In many studies, the interventions planned started from the first semester of life and were not strictly coinciding with the beginning of CF, thus making it impossible to establish a specific correlation between CFP and the precise time period of CF;
- The RCF instructions given to the caregivers in the intervention groups were part of multi-component interventions with general instructions on childcare; on the contrary, the children in the control groups were given the usual standard of care, based on some good practices that, even if in a non-pre-established way, could contain CFPs similar to those recommended in the intervention groups.
- The monitoring of caregivers’ compliance and consistency in implementing the instructions received was not always ensured and checked [6].

Despite the outcome indicators being partially different and the weaknesses of the studies, the results of the two RCTs included in this SR are consistent and suggest that RF during the CF period is effective, leading to better weight gain in the first 2 years of life [13,40].

It should, however, be noted that in the two randomized trials (of poor methodological quality) with sufficiently long follow-ups, the positive effects reported in the first two years of life in the Nourish Study were no longer detectable at 3 and 5 years [13,14,15], while in the INSIGHT study [40], these positive effects were not clinically relevant at 3 years.

The prospective observational cohort studies of both RCF and NRCF have a number of possible confounding factors, which are not always elucidated in the individual studies.

The quality of the evidence from these studies is further lowered because of the following:

- In very few cases was the same exposure studied during the CF period and in more than one cohort;
- The outcome indicators selected and the timing of the measurements varied a lot within the individual studies;
- Finally, the results for the same indicator were in some cases conflicting or significant in one study and not significant in another.

Given the number of consistent studies, one might only assume a negative effect of the restrictive style that would favor excess weight (three out of five studies) [43,51,71], and an equally negative effect of the coercive style (pressure to eat) that would favor a lower weight (three out of five studies) [50,54,71]. However, it should be emphasized that, even when the results had statistical significance, their true clinical significance has never been quantified.

Finally, it should be noted that not only the caregiver’s attitude influences the child’s behavior, but the child’s behavior and attitudes can also influence parents’ behavior [72]. This two-way variable, which occurs in all CFPs, is very difficult to establish and quantify.

### 4.2. CFPs and Risk of Choking

A feared risk of BLW is choking due to the ingestion of certain foods such as grapes, nuts, and stringy foods. This risk has been investigated in 199 children receiving BLW, 30% of them were reported to have had at least one event of “choking” when eating solid food (apple) [73]. However, the high incidence observed in this study is likely to have been due to the difficulty for caregivers to distinguish choking from gagging, a physiologic mechanism linked to the gag reflex.

The results of the studies included in this review come from observational studies (BLW) and RCTs (BLISS) of a different methodological quality. None of these studies demonstrated a significantly higher incidence of choking in both BLW- and BLISS-fed children than in conventionally fed children. Therefore, it is not possible to state that these CFPs result in an increased risk of choking at mealtimes.

Up until 24 months of age, the risk of choking is increased by typical age-related factors such as exploring the world through the mouth, inability to distinguish between edible and non-edible objects, incomplete dentition, cone-shaped airways, and poor swallowing coordination and control [17].

In a Turkish retrospective study of children undergoing bronchoscopy for suspected aspiration of a foreign body, of the patients in whom the food-borne foreign body was actually found, 80% were feeding themselves while only 14% were being assisted by a caregiver [74].

Therefore, it appears plausible to state that the most important risk factor for choking is failure to supervise the child by an adult during the meal.

No evidence was found for a cause−effect relationship between CRF or NRCF and choking events.

### 4.3. Risk of DM2, Hypertension, and Dental Caries

Regarding the effect of different CFPs in the CF period on the risk of DM2, hypertension, and caries, no evidence whatsoever was found. As a result, the different CFPs cannot be considered as risk or prevention factors for the development of these conditions later in life.

## 5. Quality of Evidence

The quality of the evidence from the RCTs included in this SR was low. The reasons for downgrading the quality of evidence were many and varied across the studies:

- BLW or BLISS studies—outcome indicators (e.g., W and L/H) often self-reported, high loss to follow-up, analysis by protocol only, inconsistent results, and single study on some questions;
- RCF studies—uncertainty about the intervention (usually part of a multi-component intervention), borderline loss to follow-up, and single study on some questions;
- For the same reasons, the methodological assessment of the observational studies, which with the GRADE method started at a low level, was also downgraded (very low quality).

## 6. Agreements and Disagreements with Other Studies or Reviews

The update provided by this SR essentially confirmed the results of previous SRs.

## 7. Limitations of the SR and Potential Bias in the Review Process

A comprehensive search strategy was adopted to search and find all relevant studies.

This SR adopted more selective inclusion criteria compared with previous SRs. Studies with high losses to follow-up and studies conducted in low-income countries and in developed countries but with different eating habits to those in Europe, such as China, were excluded.

For this reason, the number of studies included in this SR was smaller, but there was greater transferability of the results to our population of reference.

Different outcome measures were used in the studies in question, thus making it not always possible to merge the results, some of which only appeared in narrative form.

The publication bias could not be assessed because of the small number of studies included in the analyses.

## 8. Implications for Research

Future studies are certainly needed in order to evaluate the impact of different caregivers’ practices during CF. These studies should contemplate the following:

- A design including the most important confounding factors, first of all, the diverse eating styles of the families; this will allow the reliability of the results obtained, as well as their ampler transferability;
- A clear definition of the interventions (in terms of CFPs), which should be limited to the sole period of CF (i.e., 6 months to 2 years of age) and be carefully monitored over time to ensure their real and continuous application;
- A meticulous prospective and retrospective documentation of the CFPs, which infants have been exposed to, in case of observational studies;
- Strict criteria to define which categories of infants and families can be enrolled as control groups; this will avoid similar expositions in subjects pertaining to different groups, as well as differences that might influence the results (e.g., different percentages of breastfed infants between the intervention and control groups);
- An appropriate follow-up period of time, possibly of at least three years or more, to collect data on pre-defined outcomes;
- The most limited drop-off possible, even in observational studies;
- A uniform instrumental documentation of specific outcomes, namely the anthropometric ones, that should be collected by qualified healthcare professionals.

## 9. Conclusions

Based on the literature reviewed, it is not possible to state that BLW and BLISS can influence weight and height gain in children, nor can BLW and BLISS be said to have a preventive effect on the development of overweight and obesity. In addition, it cannot be ruled out that BLW leads to macro- and micro-nutrient deficits, which means that great caution should be used when proposing this CFP to families.

The results of the studies that assessed the growth and risk of obesity and overweight show that children receiving RCF have lower BMI levels in early childhood than children whose families have adopted NRCF and/or TCF.

It should also be noted that, in general terms, there are no data on the actual food intake for each CFP model. In particular, there are no studies that have comparatively assessed energy and nutrient intakes under either model, so we lack the basics/background to study the medium- and long-term effects of the various CFPs on these health outcomes.

A correlation between the different CFPs and the risk of choking has not been demonstrated. Official documents strongly recommend that, regardless of the CFP adopted, caregivers must keep a close eye on children at mealtimes and that children should not be allowed to eat while engaging in other activities such as playing or watching TV [17,55]. The acquisition by the child of relatively independent feeding skills does not mean that the caregiver’s attention should not be focused on the child at all times.

This SR was performed according to the Preferred Reporting Items for Systematic Reviews and Meta-analysis (PRISMA) guidelines [75].

## Figures and Tables

**Table 1:** Complementary feeding models.

**Baby-led weaning (BLW)** This is a complementary feeding practice that promotes the autonomy of the child by offering him/her food that is usually consumed by the family and that the child is able to handle and bring to his/her mouth on his/her own. The child is free to choose what and how much to eat with his/her hands from the food on the table. **Baby-led introduction to solids (BLISS)** A modified version of BLW that relies on the same basic principles, while recommending that at each meal the child be offered three different types of food: an iron-rich food (red meat or iron-fortified infant cereals), an energy-rich food, and a fiber-rich food such as a fruit or vegetable. **Responsive complementary feeding (RCF)** The active behavior of the child is prioritized. Food is offered only in response to cues from the child and stops when the child stops asking. Food is offered at the times, in the ways, in the textures, and in the quantities that best suit his or her level of psycho-neuro-motor and physical development as part of what the family eats, provided these foods are suitable for the infant. **Non-responsive complementary feeding (NRCF)** NRCF is characterized by a lack of reciprocity between the caregiver and child (in terms of request/response) at mealtime. The caregiver can be “overly active” (forcing, insisting, or limiting food intake), “overly passive” (to the point of becoming too tolerant of the qualitative and quantitative choices made by the child), “predominantly functional” (using food for soothing strategies), or even “completely uninvolved” and not at all interested in the child’s request or refusal of food, as in a mechanism of detachment. **Traditional complementary feeding (TCF)** In this systematic review, TCF refers to a practice in which one meal of milk (either breast milk or formula) is replaced with a solid meal, and then, later on, and over the months, a second meal is also used as a replacement. The foods, in the form of commercial or home-made baby food, are prepared specifically for the infant and initially consist of purees given in line with local eating habits, with gradual adaptation to the use of the spoon. Subsequently, cut-up foods are added. In this practice, family foods are typically introduced at around one year of age. Indicative portions are recommended by pediatricians or by other health professionals.

**Table 2:** BLW/BLISS and risk of overweight/obesity. Summary of findings for the main comparisons.

(BLW-BLISS) Compared to (Other Models of CF) in (Healthy Child, Can Influence, Either Positively or Negatively, Infant Weight−Length Gain)					
Patient or Population: (Healthy Child Aged 6–24 Months) Setting: Outpatient Intervention: (BLW-BLISS) Comparator: (Other Models of CF)					
Outcomes	Anticipated absolute effects * (95% CI)	Relative effect (95% CI)	№ of participants (studies)	Certainty of the evidence (GRADE)	Comments
Overweight/obesity risk (BLW-observational studies) (follow up: interval 18 to 78 months; evaluated with: BMI−BMIz (% obesity overweight))	388 per 1.000 (299 to 485)	189 per 1.000	OR 0.37 (0.25 to 0.55)	969 (3 observational studies) [6,7,8]	⨁◯◯◯ Very low ^a,b^
Overweight/obesity risk (BLISS-RCT) (follow up: medium 24 months; evaluated with: WHO P/L z score/BMIz (% obesity overweight))	142 per 1.000	17 per 1.000 (0 to 1.000)	RR 0.15 (0.00 to 17.79)	457 (2 RCTs) [9,10]	⨁⨁◯◯ Low ^b,c,d^

* The risk in the intervention group (and its 95% confidence interval) is based on the assumed risk in the comparison group and the relative effect of the intervention (and its 95% CI). CI: confidence interval; MD: mean difference; OR: odds ratio; RR: risk ratio. ^a^ Voluntary recruitment of mothers intending to use BLW, uncertainty in weight measurement that was entrusted to parents with an unspecified frequency, and significant loss of data during the observation period. ^b^ Loss at follow-up at 24 months = 21.4%, lack of blindness in patients, and no ITT analysis. ^c^ Low methodological quality for % loss at follow-up, lack of blindness, and no ITT analysis. ^d^ Discordant results, high heterogeneity.

**Table 3:** RCF and risk of overweight/obesity. Summary of findings for the main comparisons.

(RCF) Compared to (Other Models of CF) in [Healthy Child, in the Period 6–24 Months], Can Influence (the Development of Overweight and Obesity)						
Patient or Population (Healthy Child Aged 6–24 Months) Setting: Outpatient Intervention: (RCF) Comparator: (Other Models of CF)						
Outcomes	Anticipated absolute effects * (95% CI)		Relative effect (95% CI)	№ of participants (studies)	Certainty of the evidence (GRADE)	Comments
	Risk with [RCF]	Risk with [other models of CF]				
Risk of overweight and obesity after 2 years. RCT (follow up: 3 years; assessed with: % of overweight/obesity children)	76/1.000 (44 to 132)	185/1.000	RR 0.41 (0.24 to 0.71)	478 (2 RCTs) [16,44]	⨁⨁⨁◯ Moderate ^a,b,c^	
Risk of overweight and obesity after 13 mo. RCT (evaluated with BMIz)	DANIELS 2012. Children in the intervention group had a lower BMIz at 13 months of age than children in the control group: 0.23 ± 0.93 and 0.42 ± 0.85 (*p* = 0.01), respectively			698 (1 RCTs) [14]	⨁⨁◯◯ Low ^d,e^	

* The risk in the intervention group (and its 95% confidence interval) is based on the assumed risk in the comparison group and the relative effect of the intervention (and its 95% CI). CI: confidence interval; RR: risk ratio. ^a^ Loss to follow-up limit (20%). ^b^ One non-randomized study. ^c^ Unique study. ^d^ Performance uncertainty (performance bias): the instructions provided to the caregivers of the active groups regarding ReCF were not the only dates, but were part of a multi-component intervention, with general instructions on the overall care of children; however, no instructions or information on the nutritional aspects are described. ^e^ The interventions were initiated in times prior to the period of the CF, thus determining a condition of poor inherence (indirectness) as the effectiveness of the intervention may have been determined on a population that had not yet had reached the age of CF.

**Table 4:** NRCF and risk of overweight/obesity. Summary of findings for the main comparisons.

(NRCF) Compared to (Other Models of CF) in (Healthy Child, in the Period 6–24 Months), Can Influence, Can Influence (the Development of Overweight and Obesity)			
Patient or Population (Healthy Child Aged 6–24 Months) Setting: Outpatient Intervention: (NRCF) Comparator: (Other Models of CF)			
Outcomes	Impact	№ of participants (studies)	Certainty of the evidence (GRADE)
NRCF. Risk of overweight and obesity. Observational (follow up: interval 15 months to 20 months; assessed with:% overweight/obesity. BMIz, ΔBMI, Skinfold.)	No significant association for all comparisons (for documented exposures ≥6 months)	(4 observational studies) [19,45,46,47]	⨁◯◯◯ Very low ^a,b,c^

^a^ Risk of bias in assessing exposure in two out of three studies. ^b^ High loss at follow-up in two out of three studies. ^c^ Different parental styles evaluated, for some unique study: Pressure to eat Responsive Restriction Indulgent Laissez-faire. However, the results are generally consistent.

## Data Availability

Not applicable.

## Supplementary Materials

The following supporting information can be downloaded at https://www.mdpi.com/article/10.3390/nu14132646/s1, File S1: Literature search and screening results. File S2: Methodological assessment. File S3: Recommendations of the GLs, results of the SRS, and studies. File S4: Meta-Analysis. File S5: Summary of findings for the main comparisons.
